# Digital Marking for Toric Intraocular Lenses in a Patient with Cyclodeviation

**DOI:** 10.1155/2021/3671951

**Published:** 2021-01-12

**Authors:** Roland Seif, Jamal Bleik, Nada Jabbur

**Affiliations:** ^1^Department of Ophthalmology, Lebanese American University Medical Center, Beirut, Lebanon; ^2^Department of Ophthalmology, Clemenceau Medical Center, Beirut, Lebanon

## Abstract

Digital marking systems have been shown to be more accurate at positioning toric intraocular lenses and hence providing better visual outcomes. Patients with cyclodeviation and concomitant astigmatism undergoing toric intraocular lens placement present an extra challenge. We present a case of a patient with high astigmatism and a preexisting superior oblique palsy where using the Verion™ digital marking system proved to be an extremely valuable tool. We suggest that using this technology is especially helpful in patients with preexisting cyclodeviation and compensatory head tilt.

## 1. Introduction

Approximately 22% of patients with cataract have substantial preexisting corneal astigmatism [1]. Toric intraocular lenses (IOLs) have been adopted in the treatment of cataract in patients with preexisting corneal astigmatism, offering the patients optimum distance vision without the use of spectacles or contact lenses. To achieve excellent postoperative results with toric intraocular lenses (IOLs), precise axis alignment is essential.

Several studies have thus highlighted the value of accurate positioning of the toric IOL to the intended alignment axis, for which multiple approaches exist [2, 3].

There are several methods used to align the toric IOL at the intended axis. Most of these use a 3-step ink-marking procedure and are performed manually. New digital markers offer integrated digital guidance for accurate alignment of toric IOLs. Those work by showing the precise steep axis on the cornea relative to high-resolution reference images of the eye. This technology has the advantage of preoperative planning and intraoperative guidance in tracking the eye. Recent studies showed significantly less IOL misalignment with image-guided systems versus manual marking for toric intraocular lens alignment in cataract surgery [4].

We thought that this technology would be extremely helpful in patients with preexisting cyclodeviation undergoing toric IOL placement since they normally present a challenge for manual pre-op marking [5] and since achieving the clearest image helps restore their functional fusional amplitudes.

## 2. Case Report

A 70-year-old male, previously healthy, presented with a two-month history of blurred vision and diplopia. He denied any history of trauma. On examination, his best-corrected visual acuity (BCVA) was 20/50 in each eye. His manifest refraction was −4 + 4.25 × 50 in his right eye (OD) and −3.50 + 4.25 × 160 in his left eye (OS). Corneal astigmatism was −2.75 × 145 in OD and −3.25 × 75 in OS on biometry and topography. Intraocular pressure by applanation tonometer was 14 mmHg OU. Slit-lamp examination revealed moderate grade of nuclear sclerosis in both eyes. Retinal examination was unremarkable. His motility was full but showed a right head tilt with a 12 prism diopter (PD) of left hypertropia (LHT) in primary position that increased to 18 PD on right gaze and 18 PD on left head tilt, in addition to 7 degrees of excyclotorsion on double Maddox rod testing. This localized to a left superior oblique palsy.

The patient was interested in gaining spectacle-free visual acuity and getting rid of the diplopia. We explained to the patient that his binocular diplopia is probably caused by a decompensated left superior oblique palsy and that the expected improvement in vision after cataract and toric IOL surgery may help him regain single binocular vision. After obtaining patient's consent and explaining that he may require additional muscle surgery in case of any residual diplopia, cataract surgery was performed. Uneventful sequential cataract surgery with successful Verion™-guided toric IOL alignment was performed. One month postoperatively, the patient was satisfied and had a manifest refraction of -0.75 with BCVA of 20/25 in each eye with no diplopia.

## 3. Discussion

The Verion™ Reference Unit (Alcon Laboratories, Ft. Worth, TX) is a preoperative measurement device that captures high-resolution digital images of the patient's eye, capturing scleral vessels, limbus, and iris and pupil features. During surgery, this device projects the horizontal axis and the estimated placement axis of the toric IOL on the patient's eye, and therefore, the surgeon can accurately place the toric IOL to its preoperatively calculated axis [3]. This “fingerprint” of the eye is used throughout the procedure, allowing the surgeon to precisely position all incisions and alignment in real time according to the surgical plan. This becomes particularly important in patients with moderate to severe corneal astigmatism [4].

In addition, patients with a preexisting cyclodeviation like our patient present an additional challenge for preoperative marking because of compensatory head tilt and the variation of the axis of astigmatism between monocular and binocular conditions [5]. We hypothesized that using the Verion™ digital marker would be ideal in such patients especially that reestablishing the clearest image through cataract extraction and accurate astigmatism correction helps with regaining their binocular fusional ability and hence resolving their diplopia. Our patient is the first reported case of using this valuable technology in such challenging cases. This is another advantage of using digital toric marking that we propose utilizing whenever available.
